# Ruxolitinib and Hepatitis-B Virus Reactivation: A Clinical Concern Requiring Prophylactic Management

**DOI:** 10.12669/pjms.41.7.12301

**Published:** 2025-07

**Authors:** Ji Eun Park, Jieun Yang, Sanghoon Han

**Affiliations:** 1 Ji Eun Park, Department of Internal Medicine, Jeju National University Hospital, Jeju National University College of Medicine, Jeju City, South Korea; 2 Jieun Yang, Department of Internal Medicine, Jeju National University Hospital, Jeju National University College of Medicine, Jeju City, South Korea; 3 Sanghoon Han, Department of Hematology and Medical Oncology, Jeju National University Hospital, Jeju National University College of Medicine, Jeju City, South Korea

Ruxolitinib, a Janus kinase (JAK1/JAK2) inhibitor, is widely used for myeloproliferative neoplasms (MPNs) such as polycythemia vera and myelofibrosis. Its clinical benefits are well established, but its immunosuppressive effects raise concerns about hepatitis B virus (HBV) reactivation.^1^

Ruxolitinib impairs dendritic cells, T-lymphocytes, and natural killer cells, and reduces cytokines like IFN-γ and TNF-α, creating an environment that can facilitate HBV reactivation.^1^ This risk is highest in patients with chronic HBV infection (HBsAg positive), particularly if antiviral prophylaxis is not used.^1^,^2^ In a review, Adesola et al. found that among 58 patients with prior HBV infection, only one of 20 patients treated with ruxolitinib experienced HBV reactivation, which resolved without stopping therapy.^1^

Duan et al. analyzed 62 MPN patients with HBV history, including six with chronic HBV. Two chronic HBV patients who did not receive prophylaxis developed reactivation and hepatitis flare after ruxolitinib, while none with resolved HBV infection were affected.^2^ This highlights the importance of antiviral prophylaxis in chronic carriers. Similarly, Caocci et al. reported a primary myelofibrosis case where ruxolitinib led to HBV reactivation, emphasizing the need for careful monitoring and timely intervention.^3^

HBV reactivation can cause severe outcomes, from asymptomatic DNA elevation to fulminant hepatitis or liver failure.^2^,^3^ Reactivation may force interruption of ruxolitinib, potentially compromising disease control. International guidelines and recent reviews recommend screening for HBsAg, anti-HBc, anti-HBs, and HBV-DNA (if anti-HBc positive) before ruxolitinib, and prophylactic antiviral therapy for chronic HBV patients.^1^,^2^

Although risk is lower in resolved HBV infection, regular monitoring of liver function and HBV DNA is advised, especially in high-prevalence regions. Some reports show that stopping prophylaxis too early can result in reactivation during ruxolitinib.^3^

In summary, clinicians prescribing ruxolitinib should ensure HBV screening and appropriate prophylaxis to maximize therapeutic benefits and minimize hepatic complications.

## References

[1] Adesola AA, Cozma MA, Chen YF, Srichawla BS, Găman MA (2023). Risk of hepatitis B reactivation in patients with myeloproliferative neoplasms treated with ruxolitinib. World J Hepatol.

[2] Duan MH, Cao XX, Chang L, Zhou DB (2021). Risk of hepatitis B virus reactivation following ruxolitinib treatment in patients with myeloproliferative neoplasms. Hematology.

[3] Caocci G, Mulas O, Atzeni S (2013). Reactivation of hepatitis B virus infection following ruxolitinib treatment in a patient with primary myelofibrosis. Leukemia.

